# Immune regeneration: implications for cancer immunotherapy and beyond

**DOI:** 10.1172/JCI192731

**Published:** 2025-07-01

**Authors:** Steven L. Reiner

**Affiliations:** Department of Microbiology and Immunology and Department of Pediatrics, Herbert Irving Comprehensive Cancer Center, Columbia University Irving Medical Center, New York, New York, USA.

## Abstract

Cancer care is being transformed by therapies leveraging T lymphocytes to attack tumor cells. In parallel, recent basic discoveries have converged into a framework of lymphocyte-dependent immunity as a regenerative process that is sometimes outstripped by high-level engagement. In a stem cell–like fashion, selected T cells must balance mutually opposing demands of differentiation and self-renewal. Activating versus inhibitory signals to T cells instruct opposing cell metabolism, linked to alternative cell fates that arise in sibling cells through lopsided information transfer. Emerging studies indicate that durable immunotherapy response may be limited by the abundance of self-renewing T cells. Leveraging of basic discoveries of regenerative signaling to bolster sustained, stem-like output of freshly differentiated T cells is offering new strategies to overcome cancer immunotherapy resistance. Lymphocyte regeneration may also sustain harmful autoimmune attack. Undercutting the self-renewal of pathogenic clones may thus emerge as a therapeutic strategy for autoimmune diseases.

## Introduction

**Figure 1:**
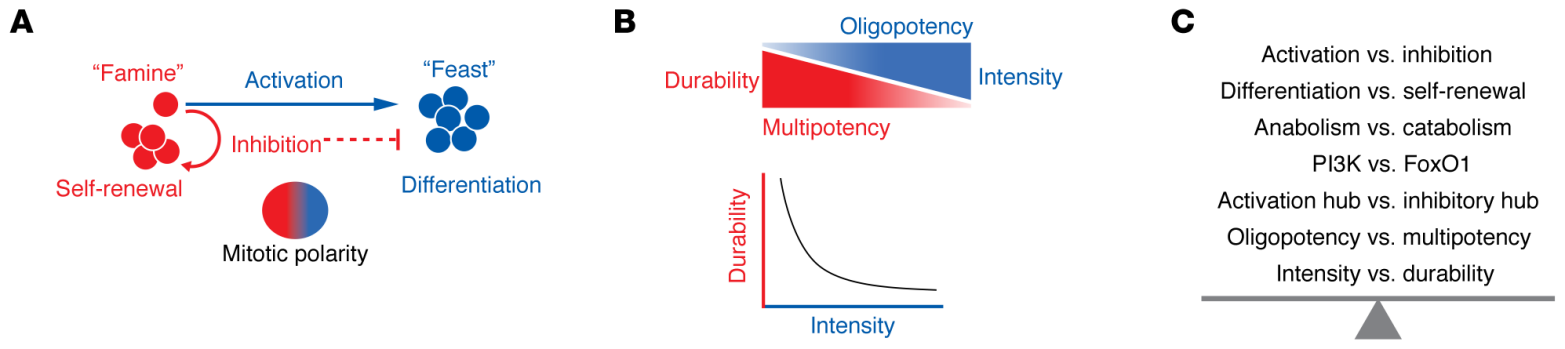
Immunity as duality of signaling, cell fate, and response dynamics. (**A**) Activating signals to T cells, through anabolic metabolism induction (“feast”), drive cell division and functional differentiation (change from red to blue). Inhibitory signals oppose anabolic induction (“famine”) and functional maturation, thereby maintaining self-renewal (red). One cell can yield opposing outcomes in its daughter cells by unequal transmission of activating and inhibitory signals during cell division (color gradient in oval mitotic cell). (**B**) Activation, division, and differentiation (blue wedge) embody response intensity and narrowing of potential (loss of self-renewal). Self-renewing cells (red wedge) reiteratively remake themselves as they yield differentiated descendants, embodying response durability and multipotency. In the lower plot, inverse relationship between response intensity and durability is a potential vulnerability of cancer immunotherapies that focus on intensifying T cell responses in the setting of imperiled T cell durability. (**C**) List of mechanistic details in the duality of signaling, cell fate, metabolism, cell biology, and response dynamics. Improving immunotherapy may require creative strategies to contort the natural regenerative balance in order to optimize intensity along with greater durability.

For more than a century, the potential role of the immune system to control cancer has been recognized (reviewed in refs. 1–3). Yet only in recent years has the immune system begun to be leveraged in a widespread manner as a pillar of cancer treatment alongside surgery, radiation, and chemotherapy. Contemporary immunotherapy breakthroughs generally harness the function of cytotoxic (killer) CD8^+^ T cells to destroy cancer cells (reviewed in refs. 1–3). The deployment of successful T cell–based platforms was ultimately enabled by the basic discoveries of a duality in the signal transduction of antigen-triggered T cells: adequacy of T cell activation is licensed by costimulatory signals that are essential cofactors of antigen receptor signaling, while maximal T cell activity is limited by inhibitory signals that serve alternative functions in immune response homeostasis and durability (4, 5) (Figure 1).

Blockade of the inhibitory receptors cytotoxic T lymphocyte antigen 4 (CTLA-4) and programmed cell death 1 (PD-1) marked the beginning of a new era in cancer immunotherapy (6–9). So powerful are the inhibitory signals in restraining complete T cell activation and function that the 2018 Nobel Prize in Physiology or Medicine celebrated James Allison and Tasuku Honjo for the “discovery of cancer therapy by inhibition of negative immune regulation” (10). Currently, blockade of the PD-1 pathway (using therapeutic antibodies that block the receptor or the ligand) is the most common cancer immunotherapy. Blockade of many other inhibitory receptors, alone or in combination with anti–PD-1, is also being evaluated. Other exciting immunotherapy approaches such as expansion and infusion of patients’ own antitumor T cells or normal T cells engineered with chimeric antigen receptors (CAR T cells) specific for tumor antigens are now being deployed for a limited set of cancers (reviewed in refs. 1–3). Therapeutic, personalized neoantigen vaccines are also being evaluated in clinical trials (11). Each of these approaches has in common CD8^+^ killer T cells as the major effector cell of tumor destruction.

Despite their promise and the advent of miraculous cures not seen with surgery, chemotherapy, and radiation, the majority of cancer patients do not achieve sustained benefit from immunotherapies such as PD-1 blockade (12). Moreover, current biomarkers do not adequately predict patient response or resistance to immunotherapy; and successful strategies to overcome immunotherapy resistance are lacking. Those gaps may reflect incomplete understanding of the mechanism of action of immunotherapy itself (1, 3). In addition, limitations in our understanding of the nature of durable T cell immunity may also be contributing to this issue (13). A common feature of immunotherapy resistance is the initial or acquired inability to mount antitumor T cell responses of sufficient intensity and duration to support elimination of the tumor burden.

The past 10 years has witnessed unexpected and seemingly disconnected basic discoveries on the nature of lymphocyte-dependent immunity coalesce into a unifying framework wherein cellular regeneration of antigen-activated lymphocytes is the central principle of adaptive immunity. These basic discoveries along with other insights have converged to refocus our understanding of the mechanisms of action of cancer immunotherapy and to provide clues to the possible cause of large swaths of immunotherapy treatment resistance. This Review will summarize the basic discoveries in lymphocyte signaling, metabolism, and cell biology that have led to the current framework of clonal regeneration (Figure 1). Emphasis will be placed on how recent discovery science is informing both new advances and remaining challenges facing cancer immunotherapy as viewed through the lens of T cell immunity as a regenerative process. In addition to the limitations in lymphocyte regenerative capacity that are faced in cancer immunotherapy resistance, new knowledge concerning the vulnerabilities of T cell regeneration will likely be leveraged in future approaches to treat autoimmunity and other destructive processes mediated by regenerating lymphocytes.

## Lymphocyte imperative: use it, but don’t lose it

**Figure 2:**
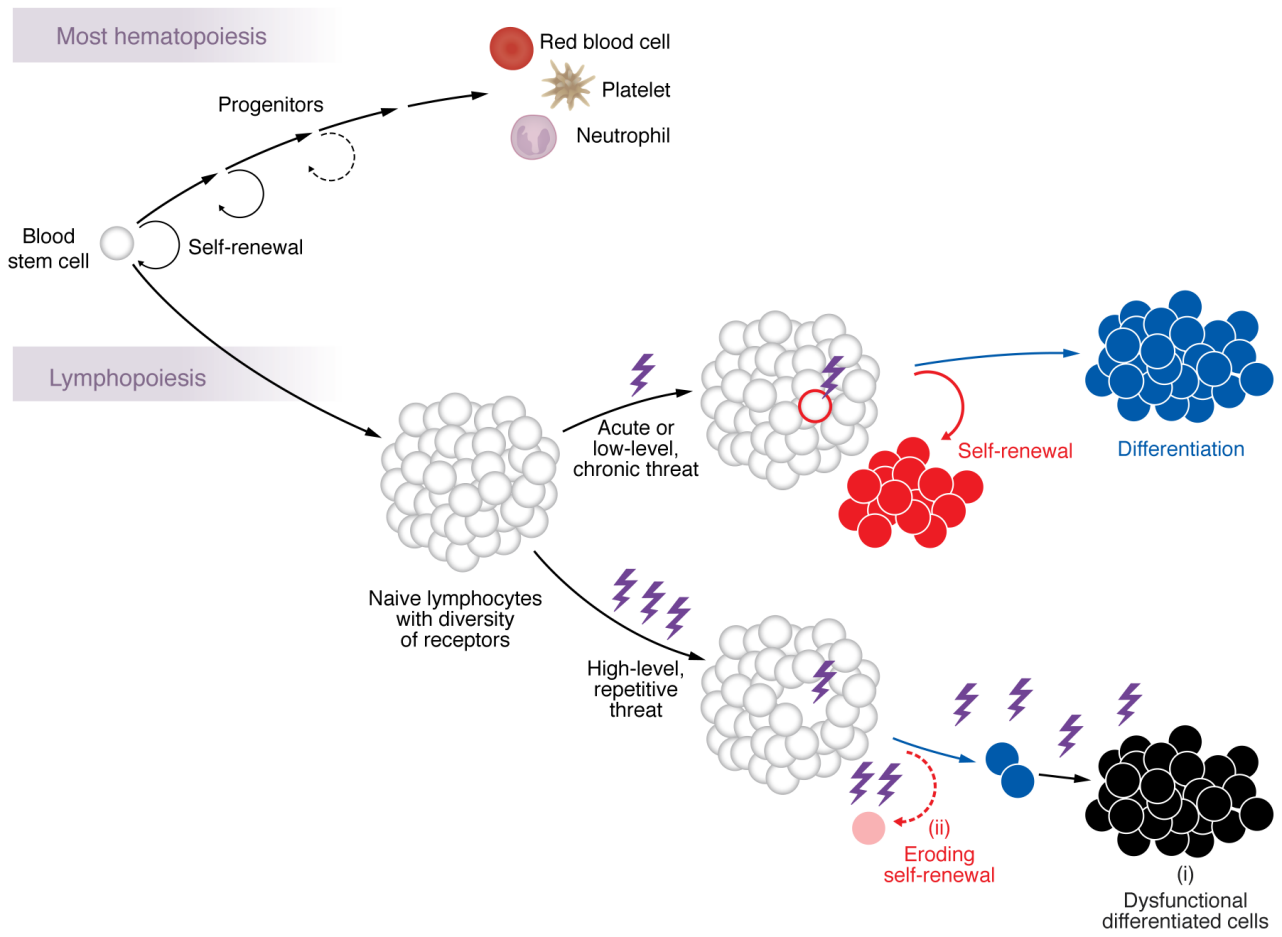
Lymphocytes engaged in immunity must regenerate, which has limits. Most hematopoiesis involves continuous production of differentiated lineages to offset continuous loss. Some capacity for facultative “tuning” of output can occur in emergencies, such as severe blood loss or infection. By contrast, lymphopoiesis generates a diverse repertoire of unique antigen receptors on individual clones (gray-outlined circles), followed by elimination of strongly autoreactive clones, and export to periphery in anticipation of immune response. In an acute or low-level persistent immune response, a sole T cell clone with correct receptor (red outline) is activated by antigenic plus costimulatory signals (collectively represented by lightning bolts), causing cell division, which is accompanied by differentiation and self-renewal by clonally related descendants of the selected cell. When the threat entails high-level, repetitive activation, at least two separable problems of failing immunity can ensue: (i) acquired dysfunction of differentiated cells, often referred to as “exhaustion,” and (ii) eroding abundance of self-renewing cells, which can result in diminished output of fresh, differentiated cells or complete loss of the clone and its descendants. It is speculated that prevention of erosion of self-renewal may be more actionable than reversal of differentiated cell dysfunction.

Naive lymphocytes recruited into immunity by virtue of their useful antigen specificity must expand in abundance and yield differentiated cell descendants, often for a lifetime (14). Unlike most other blood lineages, wherein loss of terminally differentiated cells is replaced from blood (hematopoietic) stem cells (15), lymphocytes that are consumed in the immune response face the unique requirement of clonal regeneration (16, 17) (Figure 2). A useful lymphocyte clone cannot depend on blood stem cells for replenishment owing to the fact that each lymphocyte acquires a unique and virtually irreplaceable antigen receptor sequence during its development via a process of random recombination and nucleotide insertion in the antigen receptor gene segments.

When a pathogen is completely eliminated following an acute infection, replenishment of self-renewing T cells that are clonally identical to their battle-tested kin is essential should the very same infection recur (18, 19). Because production of those less differentiated cell descendants during the primary infection typically exceeds the quantity of the original naive lymphocyte clone, preservation of the breadth of the immune repertoire is typically accompanied by enhanced depth (abundance) of the specific lymphocyte defense against the former foe. The leaving behind of more clonally identical, self-renewing lymphocytes after the useful parental cell is consumed partly explains the phenomenon of immunity, wherein elimination of secondary challenges with an acute infection is generally faster and more exuberant than the primary response (20).

The need for T cell self-renewal alongside differentiation is also of paramount importance in the event that a pathogenic threat (such as a virus or intracellular microbe) is never fully eliminated (Figure 2). Examples of such a scenario are manifest in our lifelong control of asymptomatic infections such as cytomegalovirus or the parasite *Toxoplasma gondii* (21, 22). As long as T cell–dependent immunity is intact, low-level, asymptomatic infection is maintained by continual regeneration of differentiated descendants from self-renewing kindred cells. Should T cell immunosuppression occur, the infections can disseminate “opportunistically” and cause disease. A useful, recruited lymphocyte must, therefore, be stem cell–like, able to balance two mutually opposing demands (differentiation and self-renewal) for lifelong regeneration.

## Pushing the limits of immune regeneration

In contrast with the successful regeneration of T cell defense during clearance of acute infection and during the lifelong suppression of asymptomatic low-level infections, the T cell immune response to high-level, repetitive antigen activation, most notably, chronic-active viral infections (such as untreated hepatitis C or HIV before the advent of effective antivirals) and cancer, has been regarded as a distinct situation altogether (Figure 2). Progressive increase in viral burdens or continued growth and spread of tumors often represents a failure of T cell control in the face of antigen-specific T cell responses (reviewed in ref. 23). A state of dysfunction of differentiated T cells (termed “exhaustion”) has been ascribed as the dominant problem in such scenarios. The prominent feature of dysfunctional, differentiated T cells is their expression of multiple inhibitory receptors. Recent discoveries, however, indicate that incapacitation of differentiated T cells by high-level, repetitive antigen activation may not be the sole reason for a virus or cancer to replicate uncontrollably. Additionally, dwindling regenerative capacity of self-renewing T cells is emerging as an equally critical barrier to effective viral or tumor control in such situations with less-than-optimal immunity (Figure 2).

In preclinical models of chronic-active infection and cancer, it appears that states of high-level, repetitive antigen activation can lead to wholesale loss of overactivated T cell clones (clonal deletion) as well as severe dysfunction of remaining antigen-specific, differentiated T cells (refs. 24, 25 and reviewed in refs. 23, 26–28). The maintenance of continued output of fresh effector T cell responses in the context of high-level, repetitive antigen activation is contingent on the persistence of self-renewing T cells that can regenerate differentiated cell descendants while recreating their less differentiated state, analogous to the scenarios of successful elimination of acute infections or lifelong suppression of chronic low-level infections. Self-renewing populations of T cells, which are marked by expression of the transcription factor TCF1, have been identified in acute, persistent, and chronic-active infections, as well as cancer, in humans and mice (18, 21, 22, 29–56). Across the spectrum of successful and failing scenarios of T cell–dependent control, TCF1^+^ T cells are, thus, responsible for the replenishment of freshly differentiated effector-like T cells.

If regeneration underpins the successful maintenance of high-intensity immune responses to chronic-active viral infection and tumors, then scenarios with frank loss of relevant T cell clones and/or dwindling output of fresh effector T cell descendants could be regarded as scenarios at or approaching the upper limits of lymphocyte regenerative capacity. Although still incompletely understood, some of the factors driving T cell clones past their limits of physiological capacity for self-renewal in the setting of chronic-active infections and cancer can be inferred. In addition to repetitive high-level antigen activation, increased strength of T cell receptor signaling and paucity of rest between intervals of activation apparently contribute to the loss of self-renewal capacity (27, 28, 57–60).

## Metabolic switching as framework of a regenerative duality

How are the competing outcomes of differentiation and self-renewal achieved in the course of T cell engagement? A fundamental breakthrough in our understanding of T cell activation and differentiation in the immune response arose from the recognition that signal transduction for nutrient uptake and cell growth, i.e., anabolic induction, was a hallmark event for clonal expansion of a previously quiescent and naive lymphocyte (4). Subsequently, aerobic glycolysis with lactate production, the hallmark of anabolic switching, was linked with the acquisition of effector function among the cell descendants of an activated T lymphocyte (61, 62). Specifically, activation of the phosphatidylinositol-3-kinase (PI3K) pathway downstream of combined signaling through the antigen receptor plus the costimulatory CD28 receptor licenses the switch from quiescent, catabolic metabolism to proliferative, anabolic metabolism through canonical effects on AKT and mTOR activation (4).

Subsequently, a sufficient degree of PI3K activation mediated by antigenic and costimulatory signaling is an obligatory gateway for self-renewing T and B lymphocytes to lose their capacity for self-renewal and undergo irreversible differentiation into the effector T cell and plasma cell lineages, which is marked by silencing of TCF1 and paired box 5 (Pax5), respectively (18, 31, 36, 37, 63–68) (Figure 1). PI3K-driven silencing of Pax5 and TCF1 results from the inactivation of FoxO1, which is a transcription factor that maintains Pax5 and TCF1 expression in the B and T lineages, respectively (18, 31, 63, 65, 66). Inactivation of FoxO1 is mediated by AKT activation, as a consequence of PI3K activation, underscoring the upstream role of metabolic signaling in adjusting the balance of differentiation and renewal.

As it was being discovered that anabolic induction of cell expansion is the driver of divisions, differentiation, and function, it also became evident that the metabolic strategy suitable for conditions of nutrient and energy limitation was a key requirement of quiescence and self-renewal. Catabolic metabolism, including mitochondrial respiration, fatty acid oxidation, and autophagy, was revealed to be deterministic of the quiescent and self-renewing T cell fates, particularly naive and central memory T cells (reviewed in ref. 69). At the molecular level of information transfer, activating receptors of T cells promote anabolic metabolism, while inhibitory receptors oppose anabolic induction and promote quiescent catabolic metabolism (4, 70–72). Thus, the dichotomy of signaling to lymphocyte “feast” versus “famine” directs the opposing cell fate choices of differentiation versus self-renewal, respectively (Figure 1). Repetitive, high-level T cell activation in the setting of chronic-active infections and cancer may place untenable demands on both anabolic and catabolic metabolism, thereby contributing to defects in both differentiated cell function and progenitor cell self-renewal (73, 74).

## Tripartite regeneration: two fates and an unstable intermediate

**Figure 3:**
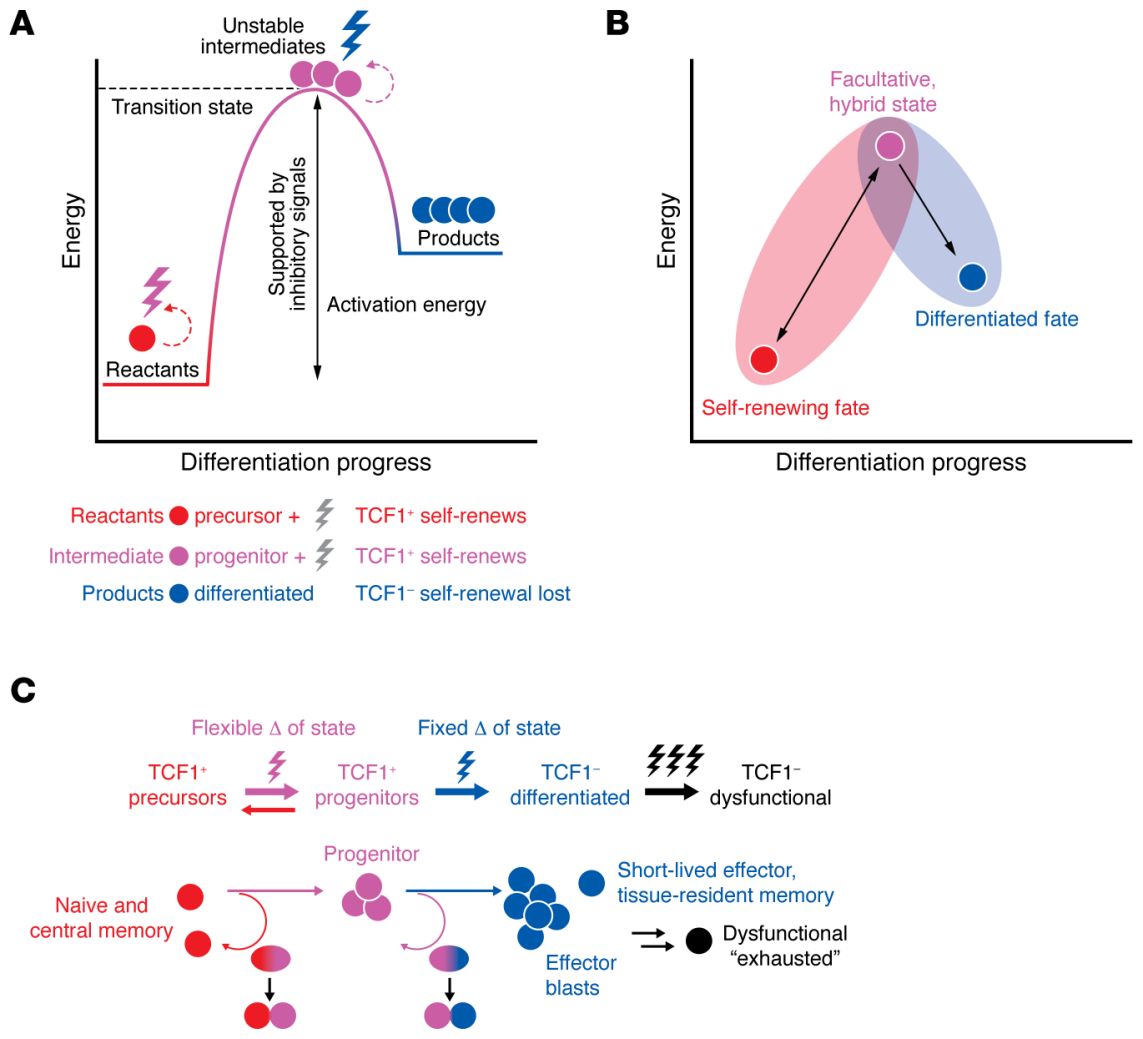
Lymphocyte differentiation stages resemble a chemical reaction. (**A**) Hypothetical reaction progress wherein reactants are quiescent precursors (red cell) at low stable energy level plus the stimuli of antigen and costimulatory signals (together represented by lightning bolt), resulting in PI3K activation, anabolic induction, and cell division with ascent of some progeny to the energetic transition state. The unstable intermediate (purple, progenitor cells) with further activation (lightning bolt) and cell division yields the product (blue, irreversibly differentiated cells), which has absorbed energy to a stable, somewhat higher level than the precursor, and silencing of TCF1 (TCF1^–^) by virtue of PI3K’s inactivation of FoxO1, an obligatory guardian of TCF1 expression. Inhibitory signals to T cells are thought to support the threshold of activation energy required for reaction progress. (**B**) The unstable intermediate (progenitor) at peak energy levels represents a facultative, hybrid state. Under continued stimulation it is anabolic, with gene expression resembling that of effector cells, yet not committed to differentiation. Upon pathogen clearance, the progenitor reverts (rolls backward downhill) to the gene expression and homing patterns of the quiescent precursor fate when there is no longer an active need to yield the product. (**C**) Schematic of three stages indicating which stages are reversible or unidirectional (top portion) and where asymmetric division results in sibling cells with opposing outcomes (bottom portion), undertaking forward progression (straight arrow) while staying in place (backward looping arrow). Asymmetric divisions begin with interphase polarity following activation (not shown), giving way to mitotic polarity (color gradient), resulting in unequal transmission of PI3K signaling during division (bicolored sibling pair). Adapted from ref. 13 with permission.

Although the stages of cellular differentiation resulting from lymphocyte activation during the immune response are occurring across the space and time of cell divisions and distances traversed in trafficking, the changes could well be understood as a process highly analogous to a chemical reaction, with three recognizable stages (Figure 3). A quiescent, self-renewing lymphocyte (such as a naive or central memory T cell) together with its activation stimuli (the ligands for its antigen and costimulatory receptors) are the reactants. The energy acquired through PI3K activation supports cell growth, division, and sufficient activation energy in some of its initial descendants to reach the transition state (highest energy level) of the reaction. The highly anabolic, great-granddaughter cell is the unstable intermediate and the direct progenitor of an irreversibly committed differentiated cell, which can be viewed as the product of the reaction.

After reactants (stimulated precursor) give rise to the unstable intermediate (progenitor), further PI3K activation yields the product of the reaction (a differentiated effector T cell), which silences TCF1 during a progenitor cell division. The TCF1-silenced (TCF1^–^) progeny will not give rise to TCF1-expressing (TCF1^+^) self-renewing cells under physiological conditions, which is analogous to the irreversible silencing of Pax5 that occurs when B cells give rise to irreversibly committed plasmablasts (18, 19, 31, 47, 52, 53, 63). Whether TCF1 silencing might be reversible in some experimental conditions is an issue that has been recently raised (75).

Nascent TCF1^–^ effector cell products are not inert postmitotic cells, but rather are capable of further division as well as function (18, 19, 42, 47, 53). In this idealized endothermic process, energy is absorbed in the stable energetic state of the TCF1^–^ effector cell product, presumably for further cell divisions, as well as the synthesis and release of cytokine and cytotoxic cargo (processes not required of the quiescent precursor/reactant). The unstable intermediate (progenitor) is a veritable hybrid stage (Figure 3). It is highly anabolic, with a gene expression program resembling effector cells while it is undergoing activation. Yet the unstable intermediate (progenitor) is not irreversibly committed to differentiation (by virtue of expressing TCF1), and it is able to revert to back to the gene expression program, catabolic metabolism, and circulatory patterns of quiescent precursors upon antigen clearance (18, 19, 47, 53). In this way, the progenitor is a facultative state contingent on the ongoing presence of antigen (Figure 3).

**Figure 4:**
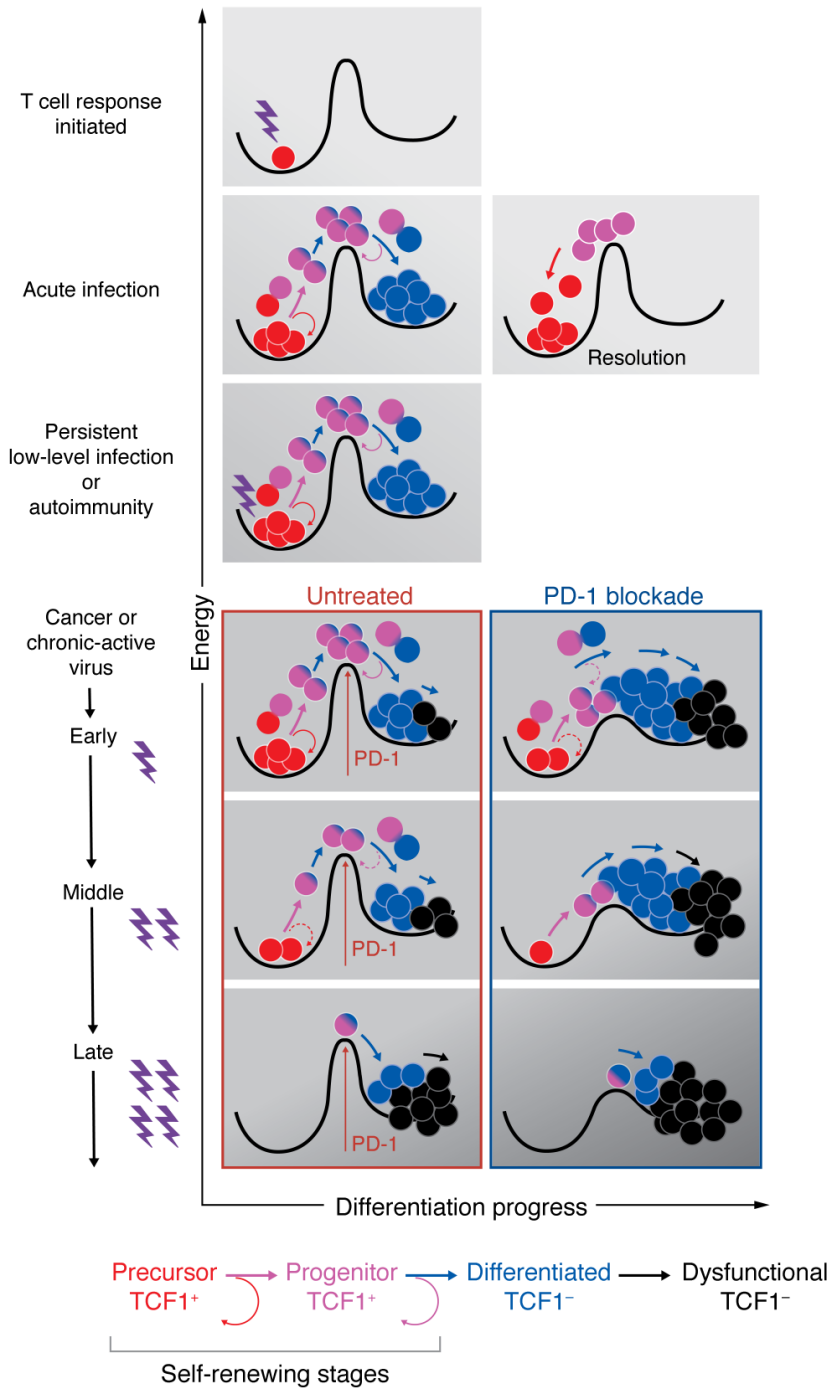
Successful and insufficient immunity from a regenerative perspective. All responses are triggered by sufficient antigen plus costimulatory signals (lightning bolt, row 1). Successful immune regeneration during acute infection and upon its resolution (row 2) illustrates the facultative (threat-dependent) nature of the progenitor and differentiated stages. Lifelong control of persistent low-level infection is analogous to perpetuation of autoimmune attack (row 3), with ongoing impetus for regeneration. Rows 4–6 depict vertical downward progression of tumor growth or increasing viral burden alongside hypothetical regenerative status without treatment (left) or with PD-1 blockade (right). The high incidence of immunotherapy resistance is probably related to progressive loss of self-renewing T cells as disease burden progresses. Optimal outcomes are more likely to be achieved when treatment is initiated earlier or with interventions that could prolong the window of self-renewing T cell abundance. Not shown is potential benefit of the arising of new neoantigens, which would appear as new red clones being deposited into the left-hand well. PD-1 blockade is depicted as lowering the activation energy and catalyzing greater division and differentiation from self-renewing cells while they persist. Adapted from ref. 134 with permission.

Recent evidence supports the view that the aforementioned tripartite sequence of cellular differentiation operates similarly across the disparate scenarios of acute and chronic stimulation (Figures 3 and 4). In both acute and chronic immune challenges, progenitor cells that can give rise to differentiated cells use a common mechanism, called asymmetric cell division, to balance the opposing demands of differentiation and self-renewal (refs. 50, 76 and discussed further below). Moreover, the progenitor T cells characteristic of chronic-active immune challenges are evident in an anticipatory manner at the outset of acute immune responses, indicating a fundamental conservation of the regenerative logic of lymphocytes (52, 53). The main variance in the setting of high-level, repetitive stimuli seems to be the erosion of self-renewal capacity and the progressive dysfunction of differentiated cells (Figures 2 and 4).

## Lopsided cell divisions: looping backward while progressing forward

As mentioned above, the proposed reaction schema is not simply progressive transformation of a single, interphase cell. It is occurring within the space and time of cell divisions. When the precursor becomes activated, it gives rise to one daughter cell that is destined for further activation and a sibling cell that remains less activated (31, 50, 64, 76–82) (Figure 3). Likewise, the unstable intermediate cell in the transition state does not itself become the product, but instead gives rise to a daughter cell during a subsequent cell division that has undergone irreversible commitment to the effector cell lineage alongside a sister cell that retains the progenitor state. In the range of murine models tested, as well as in human cells activated in vitro, the hallmark of TCF1^+^ progenitors is their ability to make differentiated TCF1^–^ progeny while also self-renewing the TCF1^+^ fate (18, 19, 30, 31, 42, 47, 50, 53, 76).

A potential solution for how lymphocytes can achieve the paradoxical, stem cell–like behavior of producing a differentiated progeny while self-renewing the less differentiated fate arises from the unique window of opportunity afforded by a cell division — to have unequal information transfer to the two daughter cells (Figure 3). When B or T lymphocytes become activated, key activating receptors that had been distributed diffusely over the surface of the cell undergo rapid reorganization to become clustered at the site of stimulation (31, 50, 64, 76–82). Such polarization of activating and adhesive receptors follows the reorientation of the microtubule and actin cytoskeleton toward the stimulatory pole, in a process termed the immunological synapse (83). There is also reorganization of inhibitory receptors and signaling molecules at the distal pole of the cell, away from the stimulatory side of the cell (76, 84). The alignment of an activating hub that promotes strong PI3K activation at one end of the mitotic spindle while an inhibitory hub forms at the other end of the spindle appears to facilitate the instruction of disparate cell fates between the two daughter cells during the cell division of an activated progenitor cell (31, 50, 64, 76, 82) (Figures 1 and 3).

In the initial T cell division(s) of the immune response, asymmetric inheritance of anabolic signaling results in a more activated daughter cell and a less activated sibling cell that resembles the reactant (which can be thought of as a quiescent precursor). The more activated descendant (progenitor) is progressing toward differentiation, but not irreversibly committed (Figure 3). It is also capable of reverting back to a precursor upon clearance of antigenic threat (removal of activation stimulus). When an activated progenitor of differentiated cells reaches the transitional state as an unstable intermediate, it becomes competent to yield an irreversibly committed, differentiated daughter cell (the reaction product) alongside a sibling cell that retains the more flexible progenitor fate. Quiescent precursors thus give rise to anabolic progenitors while maintaining the precursor. The process appears to be reversible at this stage akin to effector lineage *specification* (expression of effector genes that goes away when stimulus is withdrawn). The anabolic progenitor gives rise to the differentiated effector cell while maintaining the progenitor. The process appears to be irreversible at this stage, akin to lineage *determination* (Figure 3).

Variation in the proportions of eventual fates derived from single T cells had previously raised doubt concerning compatibility with asymmetric division models (85–87). Subsequent appreciation that the determinism of making two sibling cells different from one another is secondarily shaped by variation in antigen encounters has helped reconcile the apparent paradox (88). B cells and CD4^+^ and CD8^+^ T cells undergo asymmetric divisions in acute infections and immunizations, as well as during chronic-active viral infection and cancer models (18, 31, 37, 50, 64, 76–79, 89). CAR T cells also undergo asymmetric cell divisions in order to maintain self-renewal alongside differentiation (82).

## Rethinking the mechanism of action of immunotherapy

In parallel to the realization that regeneration is required to sustain antitumor T cell immunity, the field of cancer immunotherapy has undergone a sea change in understanding how PD-1 blockade works to intensify CD8^+^ T cell responses. Owing to the expression of multiple inhibitory receptors on the most dysfunctional of differentiated T cells, the enhanced abundance of functional cells that is observed following treatment with PD-1 blockade was initially interpreted to represent a reversal of the dysfunctional effector state. Careful analyses of mouse models as well as samples from human patients have led to the realization that, instead of reversing differentiated T cell dysfunction, PD-1 blockade is largely acting on self-renewing T cells, causing them to undergo greater proliferation and enhanced production of freshly differentiated effector T cells (22, 32, 34, 41–48, 90–92).

If activating signals are part of the reactants that propel energetic increase and forward progression, then inhibitory receptors on activated T cells seemingly function to set the height of the activation threshold needed to achieve the transition state, as well as to limit forward reaction progress toward irreversible differentiation (Figures 3 and 4). As brakes on differentiation, inhibitory signals thereby promote the preservation of self-renewing T cells, which is consistent with emerging evidence that PD-1 blockade acts to catalyze the forward reaction, manifest as enhanced cell division and more efficient output of the differentiated effector cell product, often at the expense of self-renewing T cell abundance (32, 41, 42, 45, 47, 93–97) (Figure 4).

## New perspectives on immunotherapy resistance

The realization that PD-1 blockade intensifies immune responses by driving self-renewing T cells into greater cell division and differentiation has been accompanied by a clearer understanding of why immunotherapy seems to be such a difficult needle to thread. Immunotherapy is often administered in a setting where steady-state immune response is not achieving adequate control of tumor growth. Once cancer is clinically apparent and continuing to enlarge and/or spread, the repetitive, high-intensity antigen activation of tumor-specific T cells starts to threaten the regenerative capacity of critical clones owing to a dwindling fraction of self-renewing T cells (33, 38, 45, 48, 56, 92) (Figure 4). If the obligatory focus of PD-1 blockade, i.e., the self-renewing T cell subpopulation, is becoming a vanishing target during therapy, it is perhaps not surprising that resistance to immunotherapy is common.

Several features associated with response to treatment are compatible with, albeit not definitive evidence for, a model in which PD-1 blockade may be operating within a limited kinetic window of opportunity to achieve durable clinical benefit (Figure 4): Favorable response usually associates with earlier initiation of treatment, smaller tumor burden, and increased likelihood of emerging neoantigen targets to enable sequential waves of T cell response (such as high mutational burden and microsatellite instability) (reviewed in ref. 12). PD-1 blockade in patients typically induces a monophasic burst of T cell proliferation in peripheral blood rather than ongoing expansion, even when treatments are extended beyond the peak proliferative burst (98, 99). Recent evidence suggests that addition of CTLA-4 blockade, perhaps by enhancing precursor-to-progenitor expansion, may be able to extend waves of anti–PD-1–induced proliferation (100, 101). Although PD-1 blockade intensifies division and differentiation of progenitors, it may not immediately deplete self-renewing T cell populations nor prevent the asymmetric cell divisions that yield disparately fated daughter cells (50), possibly because other inhibitory receptors, such as LAG-3, act as compensatory guardians of self-renewal (97, 102, 103).

The traditional view that immunotherapy resistance might be due to the refractory nature of the dysfunction of differentiated cells is increasingly giving way to an appreciation that erosion of regenerative capacity might be a major and actionable cause of resistance. Indeed, across immunotherapy platforms (inhibitory receptor blockade, CAR T cells, adoptive transfer of patient tumor-infiltrating T cells, therapeutic vaccines), response appears to associate with persistence of self-renewing T cells and support of the signaling pathways that maintain a balance between self-renewal and differentiation (40, 45, 48, 55, 56, 91, 92, 98, 99, 104–112).

## Prospects and predictions

The discovery of inhibitory receptor blockade has clearly changed cancer care. The recognition of inhibitory signaling, moreover, has served as a focal point for uniting seemingly unrelated basic science findings that have converged to shine a light on the “other side” of immune regeneration, the self-renewal that must accompany differentiation. Dwindling regenerative capacity because of loss of self-renewing cells can be regarded as a separable problem from previously differentiated T cells that have become dysfunctional (Figure 2), even though both problems arise from repetitive, high-level antigen activation. Rather than reversing the fate of dysfunctional, differentiated T cells, successful next-generation immunotherapy platforms and approaches to overcome resistance will need to maintain self-renewing T cell pools, the substrate for durable hyperproduction of freshly differentiated effector cells.

A candidate pathway for improving T cell durability is the metabolic switch between catabolism and anabolism, which is manifest by the activation of PI3K (driver of TCF1 silencing) leading to inactivation of FoxO1 (guardian of TCF1 expression). Studies of cells from patients with activated PI3Kδ syndrome (APDS) confirm that gain of function in the PI3K pathway can result in persistent deactivation of FoxO1 and silencing of TCF1 associated with loss of self-renewing T cells (68). Gain of function in the PI3K pathway renders patients with APDS unable to control persistent low-level infections such as CMV and EBV, which is compatible with the central premise that lymphocyte immunity depends on regeneration of selected clones. Recent T cell mutagenesis screens have also confirmed the importance of the PI3K pathway for T cell function (113, 114). Dampening PI3K activation can improve immunotherapy durability (56, 67, 109, 115–120). Enforced expression of TCF1 may also promote self-renewal (121, 122) but may not be as effective as enforced activation of FoxO1 (106, 107).

Other recent metabolic and signaling interventions that have improved immunotherapy outcomes have been linked to dampening of anabolic induction, limiting of inflammatory signaling intensity, and promoting of T cell self-renewal (108, 110, 123–128). This is compatible with a strength-of-signal hypothesis wherein greater strength of activation may push intensity and greater restraint or inhibition may promote durability insofar as the persistence of self-renewing T cells seems to be negatively influenced by the strength of interaction between T cell receptor and antigen (peptide/MHC) (24, 25, 57, 58).

The past several years has seen an explosion in our understanding of how selected lymphocyte clones manage a lifelong balancing act of differentiation and self-renewal. More recently, it has become apparent that perpetuation of autoimmunity, inflammatory bowel disease, and anti-transplantation responses may be underpinned by the principle of a regenerative duality of T cells (129–133) (Figure 4). It will, therefore, not be surprising if new treatment strategies against inflammatory attack involve the intentional burnout of self-renewing cells in order to vanquish regeneration of pathogenic clones.

## Version 1 07/01/2025

Electronic publication
